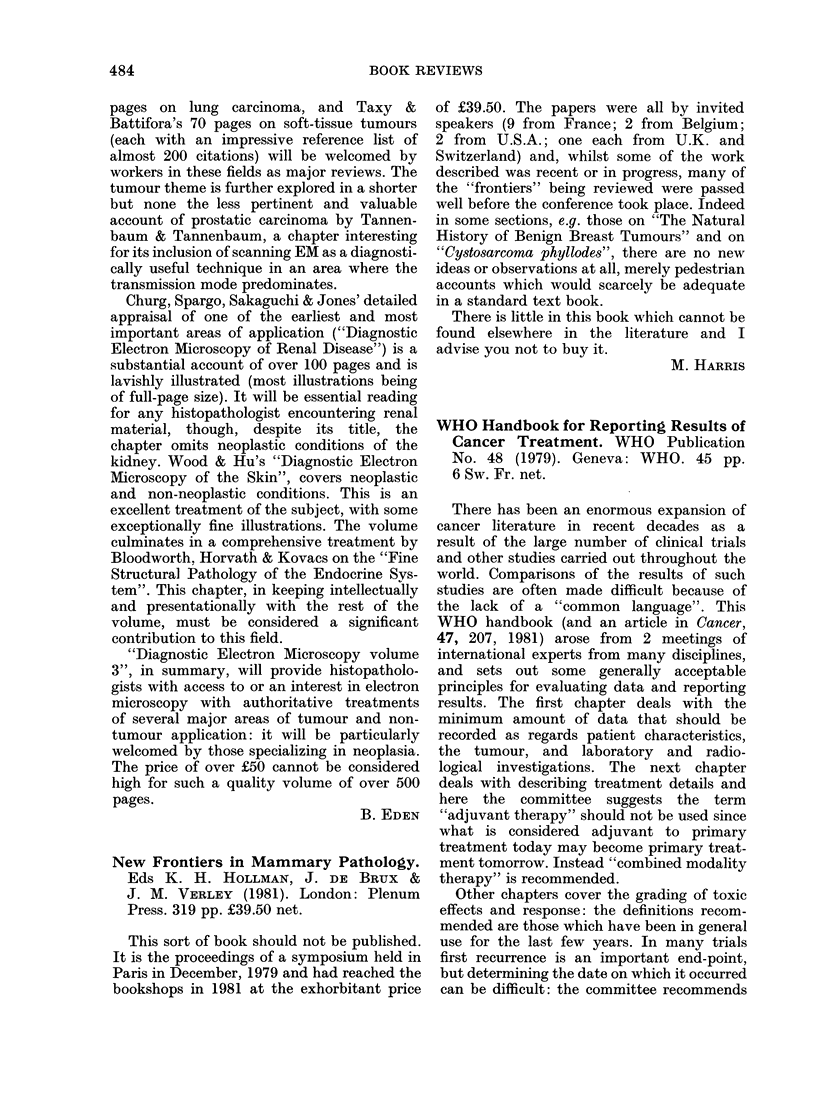# New Frontiers in Mammary Pathology

**Published:** 1982-03

**Authors:** M. Harris


**New Frontiers in Mammary Pathology.** Eds K. H. Hollman, J. de Brux & J. M. Verley (1981). London: Plenum Press. 319 pp. £39.50 net.

This sort of book should not be published. It is the proceedings of a symposium held in Paris in December, 1979 and had reached the bookshops in 1981 at the exhorbitant price of £39.50. The papers were all by invited speakers (9 from France; 2 from Belgium; 2 from U.S.A.; one each from U.K. and Switzerland) and, whilst some of the work described was recent or in progress, many of the "frontiers" being reviewed were passed well before the conference took place. Indeed in some sections, *e.g.* those on "The Natural History of Benign Breast Tumours" and on "*Cystosarcoma phyllodes*", there are no new ideas or observations at all, merely pedestrian accounts which would scarcely be adequate in a standard text book.

There is little in this book which cannot be found elsewhere in the literature and I advise you not to buy it.

M. Harris